# Supplementary material for: Patterns of Intron Gain and Loss in Fungi
Source: PLoS Biol. 2004 Nov 30;2(12):e422. doi: 10.1371/journal.pbio.0020422 (PMC532390; doi:10.1371/journal.pbio.0020422)
Supplement: Table S1 — Also available at http://genes.mit.edu/NielsenEtAl/. (4.3 MB ZIP). [file pbio.0020422.st001.zip › NielsenEtAl/html/822.html]

AN1859.1.NCU00359.1.MG05178.1.FG06737.1


```
 CLUSTAL W (1.82) Multiple Sequence Alignments - Introns Inserted


Sequence 1: NCU00359.1	309 aa
Sequence 2: MG05178.1	263 aa
Sequence 3: FG06737.1	268 aa
Sequence 4: AN1859.1	275 aa
Alignment Length: 339 aa
Number Identitical Residues: 82 aa
Alignment Score (without introns) 5025


MG05178.1 	MAPQNNLGKRVK~GTSVHRPFI~YGTTAKPFDDEKNPKPPGVPDDHTHSWQVFVKGVEDT
NCU00359.1	MAPPT--GKRVK~GVQIFRPFV~YGTTARPFDEKTNPKPPGIPDDHTHSWTVFIKGIDDV
FG06737.1 	MAPQNQLGKRVK~LTQVRRPFI~VGSTAKPFSD-TNPRPAGIPDNHTHSWQVFVKGLDDT
AN1859.1  	-MPSATGTKRVR0GVSIFRPFV1FGSEAQPFDP--ATKPSNVSSDHTHQWRVYVRGVNGE
          	  *     ***:  ..: ***:  *: *:**.    .:*..:..:***.* *:::*::. 

MG05178.1 	DVTYWLKRVQFKLHESIPNHIRM1VDSEPGKAFVVNETGWGEFEIAIKLYYVPESGEKPQ
NCU00359.1	DITYWLRRVQFKLHESIPNHVRM1VEGVKGQPFQIHETGWGEFEITMKLYYVPESSEKPQ
FG06737.1 	DITYWLRRVQFKLHESIPNYVRM1VEGEPGKPFTVEETGWGEFDITIKLYYVNDSGEKPQ
AN1859.1  	DISYWIKKVQFKLHETYVQNVRT~VEHP---PYEVTETGWGEFEIQIKIYFVPESMEKPQ
          	*::**:::*******:  : :*  *:     .: : *******:* :*:*:* :* ****

MG05178.1 	TLYHHLRLHPYGRTDAEKDAMRASG-EVTAWTYEEQIFNEPYEVFYDVLTSGALPTTAAG
NCU00359.1	TLYHHLRLHPFGRTEEEKEAMRLNGGEVISWVYEEQIFNEPYEPFYDILISGALPPSAST
FG06737.1 	TLYHYLRLHPYGRNEEEKQAMISSNGEICSWSYEEQLFNEPYEVFYNLLTQGAIP-KGWK
AN1859.1  	TLWHSLKLHPYGPDAEGK---KERREVVVSQNYEEVVFNEPVEQFYDYLTGGSGTQQMQK
          	**:* *:***:*     *         : :  *** :**** * **: *  *: .     

MG05178.1 	ANRGKG--------------KGSAPAVGG-------------------------------
NCU00359.1	LKDGGGGGGSGAATPTSANTPGASSSKGGNKKGHERSHSRASVSTNKDGKNNNESKEEPF
FG06737.1 	PSGGKG--------------KGKTRAPPP-------------------------------
AN1859.1  	GKSGKN-------------AKQAQQQRGG-------------------------------
          	 . * .             :                                        

MG05178.1 	--VRTEGGVLERSAMIPLSNRPGQPFSRDTEQLEVERLKKAQEKAQALMSTLQTQLKNKE
NCU00359.1	VIQKSEGGVLERSAMIPLVNRPGQPFSRETEQLEIQKLKEAKLKVDEMKKQMMEELEKKQ
FG06737.1 	-FPTDNNEVWEHSAMIPAHNRPGQPFSLETEAAEIRKLAEAQGQAEDMAKKILAELKAKE
AN1859.1  	-----------RTAEIPFNETPENPYSRTAENKELDRLAEANKTVEQMIKDEKERLIARE
          	           ::* **  : * :*:*  :*  *: :* :*:  .: : .    .*  ::

MG05178.1 	AALQKLRK-QTASAK-----~----------------------
NCU00359.1	KRLEALRA-ESAKAP-----~----------------------
FG06737.1 	EQLATLKG-ENQAAAAAAKS~----------------------
AN1859.1  	KRLAELRASEGVPAQPLKKS2FSGRIPVHSQQRMAGTVGPPDS
          	  *  *: ::   * .  .:  :.  .  :..  :.: ....:
```
